# Evaluation of Copanlisib in Combination with Eribulin in Triple-negative Breast Cancer Patient-derived Xenograft Models

**DOI:** 10.1158/2767-9764.CRC-24-0047

**Published:** 2024-06-05

**Authors:** Zhanfang Guo, Jingqin Luo, R. Jay Mashl, Jeremy Hoog, Piyush Maiti, Nikki Fettig, Sherri R. Davies, Rebecca Aft, Jason M. Held, Ramaswamy Govindan, Li Ding, Shunqiang Li, Cornelius von Morze, Gerburg M. Wulf, Kooresh I. Shoghi, Cynthia X. Ma

**Affiliations:** 1 Division of Oncology, Department of Medicine, Washington University School of Medicine, St. Louis, Missouri.; 2 Division of Public Health Science, Siteman Cancer Center Biostatistics Core, Washington University School of Medicine, St. Louis, Missouri.; 3 Department of Medicine, McDonnell Genome Institute, Siteman Cancer Center, Washington University School of Medicine, St. Louis, Missouri.; 4 Mallinckrodt Institute of Radiology, St. Louis, Missouri.; 5 Department of Surgery, Washington University School of Medicine, St. Louis, Missouri.; 6 Department of Medicine and Cancer Research Institute, Beth Israel Deaconess Medical Center, Boston, Massachusetts.

## Abstract

The PI3K pathway regulates essential cellular functions and promotes chemotherapy resistance. Activation of PI3K pathway signaling is commonly observed in triple-negative breast cancer (TNBC). However previous studies that combined PI3K pathway inhibitors with taxane regimens have yielded inconsistent results. We therefore set out to examine whether the combination of copanlisib, a clinical grade pan-PI3K inhibitor, and eribulin, an antimitotic chemotherapy approved for taxane-resistant metastatic breast cancer, improves the antitumor effect in TNBC. A panel of eight TNBC patient-derived xenograft (PDX) models was tested for tumor growth response to copanlisib and eribulin, alone or in combination. Treatment-induced signaling changes were examined by reverse phase protein array, immunohistochemistry (IHC) and ^18^F-fluorodeoxyglucose PET (^18^F-FDG PET). Compared with each drug alone, the combination of eribulin and copanlisib led to enhanced tumor growth inhibition, which was observed in both eribulin-sensitive and -resistant TNBC PDX models, regardless of PI3K pathway alterations or PTEN status. Copanlisib reduced PI3K signaling and enhanced eribulin-induced mitotic arrest. The combination enhanced induction of apoptosis compared with each drug alone. Interestingly, eribulin upregulated PI3K pathway signaling in PDX tumors, as demonstrated by increased tracer uptake by ^18^F-FDG PET scan and AKT phosphorylation by IHC. These changes were inhibited by the addition of copanlisib. These data support further clinical development for the combination of copanlisib and eribulin and led to a phase I/II trial of copanlisib and eribulin in patients with metastatic TNBC.

**Significance:**

In this research, we demonstrated that the pan-PI3K inhibitor copanlisib enhanced the cytotoxicity of eribulin in a panel of TNBC PDX models. The improved tumor growth inhibition was irrespective of PI3K pathway alteration and was corroborated by the enhanced mitotic arrest and apoptotic induction observed in PDX tumors after combination therapy compared with each drug alone. These data provide the preclinical rationale for the clinical testing in TNBC.

## Introduction

The PI3K pathway signaling plays a crucial role in many biological processes, including cell survival, growth, metabolism, differentiation, motility, genomic instability, and angiogenesis (1). Aberrant PI3K/AKT pathway activation has been frequently observed in triple-negative breast cancer (TNBC; refs. 2, 3). Per The Cancer Genome Atlas TNBC dataset, activation of this key pathway occurs by various mechanisms, including *PIK3CA* mutations (7%), and more frequently, deficiency or loss of expression of the tumor suppressor phosphatase and tensin homolog PTEN (35%; refs. 4, 5). Significantly, higher levels of phosphorylated AKT have been observed in TNBC compared with non-TNBC (2). In addition, PI3K pathway activation has also been linked to chemotherapy resistance (6). Various preclinical models have illustrated that inhibition of the PI3K pathway signaling enhances and synergizes with the cytotoxicity of a variety of chemotherapy agents (7–9). Increasing evidence indicates that activation of the PI3K/AKT pathway also helps maintain the stemness and chemoresistance of breast cancer stem cells (CSC; refs. 10, 11). Previous studies have shown that PI3K inhibition sensitizes these CSCs to chemotherapy and molecular targeted therapy in several cancers, including breast cancer (12). Therefore, the combination of a PI3K inhibitor with chemotherapy is an attractive therapeutic strategy for TNBC.

Eribulin mesylate (Halaven, Eisai Inc) is a non-taxane inhibitor of microtubule dynamics that is approved for the treatment of metastatic breast cancers previously treated with anthracycline and taxane (13). In the phase III EMBRACE study, eribulin mesylate was associated with significant improvement in overall survival (OS) compared with treatment of physician's choice in this heavily pretreated patient population (14). The efficacy of eribulin mesylate in TNBC was demonstrated in the pooled analysis of two phase III studies (EMBRACE/Study 305), which reported a 4.7-month improvement in median survival with eribulin compared with control chemotherapy (median OS: 12.9 vs. 8.2 months; HR 0.74; *P* = 0.006; ref. 15). In addition to the induction of an irreversible mitotic block, eribulin has been shown to impact tumor vascular remodeling (16) and inhibition of epithelial-to-mesenchymal transition (EMT) and metastasis in experimental models (17). Our group previously demonstrated that the PI3K inhibitor BKM120 enhanced the antitumor effect of eribulin in TNBC both *in vitro* and *in vivo* using PDX models (18), synergistically reducing markers of EMT and stem cells, and enhancing both mitotic arrest and apoptosis. However, BKM120 is no longer in clinical development. Copanlisib is a potent pan-class I PI3K inhibitor with activity predominantly against the α and δ PI3K isoforms (IC_50_ 0.5 and 0.7 nmol/L, respectively) compared with the β or γ isoform (IC_50_ 3.7 and 6.4 nmol/L, respectively; ref. 19). It was initially approved by the FDA for relapsed/refractory follicular lymphoma. It also demonstrated single-agent activity in solid tumor malignancies, including breast cancer, in the initial phase I study (20), with activity observed in tumors with either *PIK3CA* mutation or PTEN loss. The activity of copanlisib has not been investigated in TNBC, consequently we conducted this preclinical study to examine its antitumor activity as a single agent and in combination with eribulin in both eribulin-sensitive and -resistant TNBC PDX models.

## Materials and Methods

### Chemicals and Antibodies

Copanlisib (BAY 80-6946) and eribulin were obtained through material transfer agreement from NCI CTEP. The primary antibodies against PTEN (catalog no. 9559), Cleaved PARP (Asp214; Human Specific catalog no. 9541S), and phospho-AKTS473 (catalog no. 4060) in IHC analyses were purchased from Cell Signaling Technology, and pHistone H3 (Ser 10; catalog no. 06-570) from Millipore.

### Generation of PDX Models

Fresh tumor specimens were obtained via biopsy or tumor resection from patients with breast cancer after obtaining written informed consent, in compliance with NIH regulation, institutional guidelines and Institutional Review Board approval at Washington University (St. Louis, MO; ref. 21). Procedures for sample processing and establishment of orthotopic xenograft models in the fourth mammary fat pad of NOD/SCID mice have been described in detail previously (21). Written informed consent was obtained from patients for PDX engraftment and that PDX models are available through the application to the Human and Mouse-Linked Evaluation of Tumors core (http://digitalcommons.wustl.edu/hamlet).

### IHC

IHC analyses for PTEN, pAKT, pHistone H3, and cleaved PARP were performed on 5 µm sections of formalin-fixed paraffin-embedded (FFPE) PDX tumors as described in our previous publication (18, 22).

### mRNA Gene Expression Analysis Using Agilent 4 × 44K Arrays

RNA was extracted from cryopulverized PDX tumor tissue according to established protocol (23). Purified total RNA samples were then profiled using 4 × 44K human oligo microarrays (Agilent Technologies) as described previously. Raw Agilent 4 × 44K gene expression data were preprocess and normalized using the BioConductor “limma” package sequentially through background subtraction, loess within-array normalization and quantile between-array normalization. The probe level gene expression data were collapsed to gene level by median expression. ComBat was applied using the R package “sva” to the normalized gene expression data to correct for potential human/mouse batch effect (24). Research use only PAM50 subtype classification were described previously (25). Microarray data are available through the Gene Expression Omnibus database (GSE243865). TNBC subtypes were assigned for each of the samples using “TNBCtype” (refs. 26–28; http://cbc.mc.vanderbilt.edu/tnbc/index.php).

### Whole-exome Sequencing Analysis

The whole-exome sequencing data generation and variant calling for PDX models (WHIMs 3, 4, 6, 10, 12, 29, 34, 52) were published previously (21, 29–31).

### Reverse Phase Protein Array

Reverse phase protein array (RPPA) of PDX tumor lysates was performed at the Antibody-based Proteomics Core at the MD Anderson Cancer Center. Please see the link for a complete list of validated antibodies: https://www.mdanderson.org/research/research-resources/core-facilities/functional-proteomics-rppa-core.html.

### *In Vivo* Studies of Drug Response in PDX Models

Eight TNBC PDX models (WHIMs 3, 4, 6, 10, 12, 29, 34, 52) with varying degrees of baseline PI3K pathway activities were selected from the Washington University Human in Mouse (WHIM) PDX collection (21). Early passages of each PDX model were propagated into the fourth mammary fat pad of 6-week-old NU/J homozygous female mice (Jackson Lab, catalog no. 2019). Treatment was initiated when the xenograft tumor reached approximately 5–7 mm in diameter.

To screen for eribulin sensitivity, tumor-bearing mice engrafted with each PDX model were randomized to receive eribulin (1 mg/kg in all models and 0.3 mg/kg in selected models) or vehicle intraperitoneal weekly × 3 (*n* = 2 each arm). Bidimensional tumor measurements were performed every 2–3 days. Tumor volume (*V*) was calculated from the equation *V* = *ab*^2^/2, where *a* and *b* are tumor length and width (in millimeters), respectively.

Tumor growth and long-term survival in response to 4 weeks of treatments with eribulin and copanlisib, either alone or in combination, were examined and compared for WHIM29 and WHIM34. Tumor-bearing mice were randomly assigned to four treatment groups (*n* = 5 mice per treatment group, two tumors per mouse, one each at the left or right fourth mammary fat pad) when tumor volume reaches approximately 150 mm^3^ to receive either vehicle, eribulin (0.1–0.3 mg/kg, i.p., day 1 each week), copanlisib (10 mg/kg, i.v., days 2 and 3 each week), or the combination of eribulin and copanlisib using the same dosing and schedule for each drug, for 4 weeks, then continued to be followed until endpoint, defined as death or tumor's longest diameter reaching a maximum of 2 cm). Tumor measurements were performed every 2–3 days until endpoints were reached.

Tumor growth response to eribulin in combination with copanlisib was also screened in the rest of the six TNBC PDX models (WHIM3, WHIM4, WHIM10, WHIM6, WHIM12, and WHIM52), besides WHIM29 and WHIM34. Mice engrafted with each PDX model were randomly assigned to four treatment groups (*n* = 2 mice per treatment group, two tumors per mouse, one each at the left or right fourth mammary fat pad) when tumor size reached approximately 150 mm^3^ to receive either vehicle, or eribulin (0.2 to 1 mg/kg, i.p., day 1 each week), copanlisib (10 mg/kg, i.v., days 2 and 3 each week), or the combination of eribulin and copanlisib for 3–4 weeks. Tumor measurements were performed every 2–3 days until endpoints were reached.

To examine treatment-induced molecular changes, tumor bearing mice were sacrificed 2 hours after the final drug dosing at the completion of 3–4 weeks of treatment as described above with either vehicle, eribulin, copanlisib, or the combination, for each of the eight PDX models. Xenografts were harvested immediately, cut into pieces with some fixed in 10% formalin and some snap frozen for RPPA.

### Preclinical Fluorodeoxyglucose PET Imaging for WHIM29 and WHIM34

Metabolic fluorodeoxyglucose PET (FDG-PET) imaging was performed at baseline (prior to therapy) for tumor-bearing mice (*n* = 15 mice, each harboring two tumors engrafted at the left and the right fourth mammary fat pad) when xenograft tumor reached approximately 7–8 mm in diameter. These 15 mice were then randomized to three treatment groups (*n* = 5 per group) to receive treatment with either vehicle, copanlisib (10 mg/kg i.v., on day 1, and on day 2), eribulin (0.2 or 0.3 mg/kg i.p., on day 1), or the combination of eribulin and copanlisib with the same schedule. A repeat FDG-PET metabolic imaging was performed on day 2, approximately 4 hours after drug/vehicle administration. Mice were sacrificed on day 3 and xenograft tumors were harvested, with tumor pieces fixed in 10% formalin for FFPE block for IHC analysis of pAKT and cleaved PARP.

In preparation for FDG-PET imaging, mice were deprived of food for 4 hours prior to the imaging study. Prior to imaging, mice were anesthetized with 1%–3% isoflurane/oxygen and maintained under anesthesia with 1%–1.5% isoflurane/oxygen throughout the imaging experiment. FDG (8–12 MBq) was injected via a tail vain. Fifty-minute following FDG administration, a static 10-minute PET image acquisition was performed on the Mediso nanoScan PET/CT. Mice were maintained at 37°C during the study in a specialized temperature-controlled bed chamber. CT images were acquired prior to PET imaging for attenuation correction and anatomic co-registration. PET images were reconstructed following manufacturer's recommendations using the OSEM algorithm. PET data were analyzed manually by drawing three-dimensional regions of interest (ROI) over the tumor identified on the PET studies with correlation to CT to confirm tumor location. The uptake data were expressed as mean standardized uptake values (SUV) for each ROI at 50–60 minutes after injection of FDG.

### Statistical Analysis

LIMMA was applied to the RPPA data across PDX models, including the effect of treatment and PDX, to identify the proteins differentiating treatment groups (32). The percent change of a protein resulted from drug treatment was calculated as the difference between the estimated group means of drug-treated samples and vehicle-treated samples, divided by the absolute value of the group mean of the vehicle-treated samples. Significant differentially expressed proteins from LIMMA analyses were claimed at a Benjamini–Hochberg FDR-adjusted *P* value <5%. Tumor cells staining positive for cleaved PARP were counted in three randomly selected fields per tumor at 600× magnification and approximately 309 to 505 cells were counted. ANOVA for repeated measures was applied to model the tumor growth data and survival data, which were analyzed by using the Kaplan–Meier method and graphed in Prism, and pairwise treatment group differences were tested using the log-rank test. *P* < 0.05 was used for significance for all reported statistics in tumor growth and survival data analyses.

### Data Availability

The data generated in this study are available within the article and its Supplementary Data. Materials and models as well as RPPA data files are available upon request from the corresponding author.

## Results

### Characteristics of TNBC PDX Models

**FIGURE 1:**
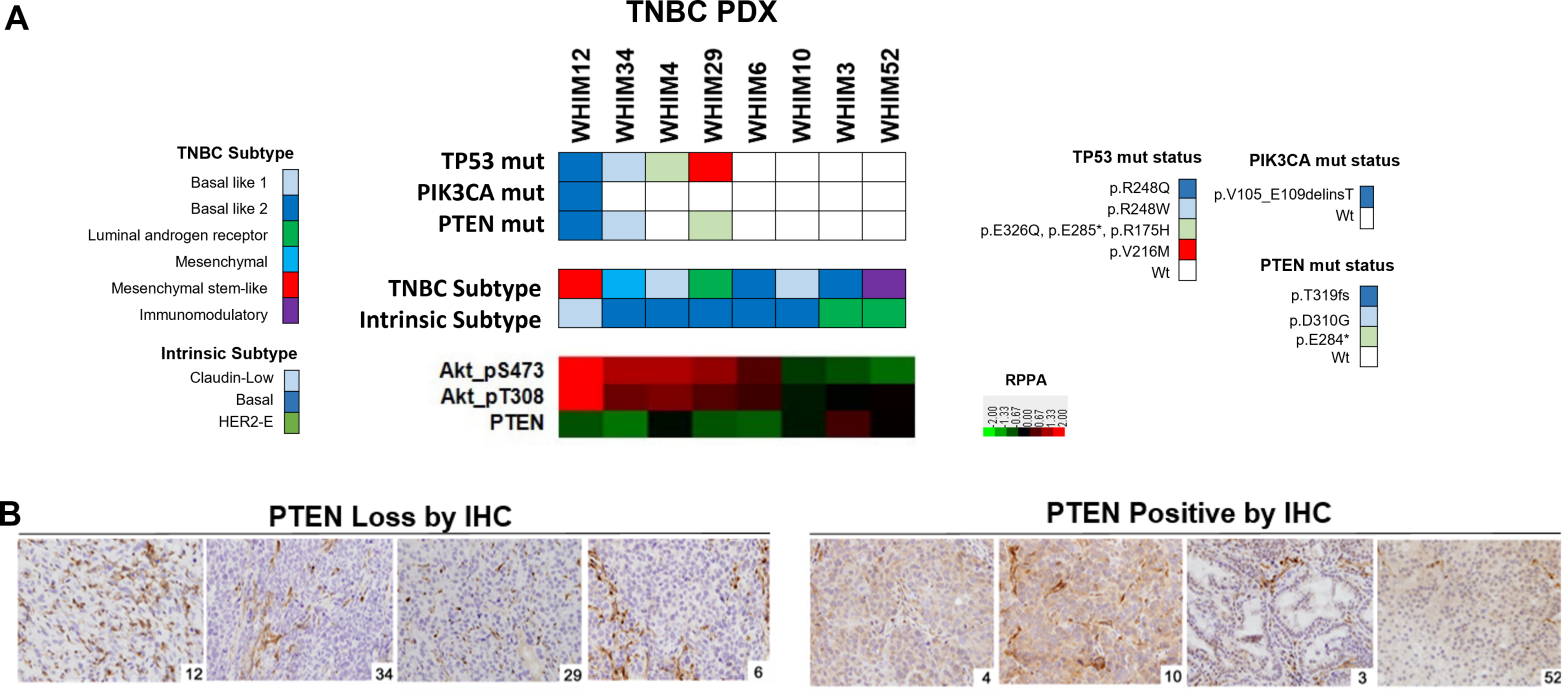
PI3K pathway alterations of selected TNBC PDX models. **A,** Molecular characteristics of the eight TNBC PDX models included in this study, including mutation status of candidate genes based on whole exome sequencing, breast cancer intrinsic subtype and TNBC subtype derived from gene expression profiling, and PI3K pathway signaling activity indicated by levels of AKT phosphorylation and PTEN by RPPA analysis for each model. **B,** Representative pictures of PTEN IHC, demonstrating loss of PTEN expression in four of the eight PDX models. The number at the right lower corner of each IHC picture refers to the WHIM ID of the PDX model analyzed. Note the positive PTEN staining of the stromal cells, which serves as an internal control.

To investigate TNBC responses to copanlisib and eribulin, we selected eight TNBC PDX models from the Washington University PDX core that vary in PI3K pathway activity (Fig. 1). All of these models have been reported previously, except the gene expression microarray data for WHIM52 (21, 29–31). Supplementary Table S1 lists the clinical characteristics and treatment history of patients before and after providing the samples for PDX engraftment. Two models were derived from African American women. All eight models were derived from patients with lethal TNBC that eventually claimed their lives. All PDX models were from biopsies that confirmed TNBC, although the patient who provided the sample establishing WHIM4 had initially HER2-positive disease prior to recurrence, and the patient who provided the sample for establishing WHIM52 initially had estrogen receptor (ER)-positive/HER2-negative disease. Five PDX models were derived from treatment-naïve tumor biopsies from patients with locally advanced (WHIM12), initial metastatic recurrence (WHIM34) or *de novo* stage IV disease (WHIMs 3, 6, 29). WHIMs 4, 10, and 52 were derived from metastatic disease relapsed after prior neoadjuvant or adjuvant anthracycline and taxane therapy.

In previous reports, we demonstrated that PDX models preserve the genomic alterations and mRNA expression profiles of the original tumor, and were genomically and proteomically stable across early passages (21). In addition, these PDX models represent the diverse intertumor heterogeneity of human TNBC in gene expression and proteomic profiles (21, 30). The eight PDX models represent a variety of TNBC subtypes and PAM50 subtypes by gene expression profiling (Fig. 1A). Supplementary Table S2 lists selected potential actionable mutations and genomic alterations in the PI3K pathway genes. These PDX models were further characterized by RPPA analysis of 380 protein markers (Fig. 1A), and IHC for PTEN (Fig. 1B). Figure 1 illustrates select PI3K pathway genomic and protein-level alterations identified in the PDX models. PTEN loss by IHC correlated with low PTEN levels on RPPA, and was observed in four PDX models including WHIM29 (*PTEN* mutations) and WHIM34 (*PTEN* mutations), WHIM6 (*PTEN* WT), and WHIM12 (*PTEN* mutations; Fig. 1; Supplementary Table S2). PIK3CA amplification was found in WHIM4. WHIMs 3, 6, 10, and 52 harbored no notable mutations in *PI3K/AKT/PTEN* or *TP53* (Fig. 1; Supplementary Table S2).

### Assessing Sensitivity to Eribulin in TNBC PDX Models

To screen the eight PDX models for eribulin sensitivity, tumor-bearing mice for each PDX model were randomized to receive either vehicle or eribulin. *N* = 2 per treatment group was justified on the basis of the feasibility and reproducibility of using 1 × 1 × 1 (1 animal per model per treatment) design for PDX oncology drug screening reported by Gao and colleagues (33). We anticipate an increase in precision by including 2 mice per treatment group and the inclusion of a vehicle treatment group.

A maximum of 1 mg/kg i.p. weekly dose of eribulin was tested in the PDX models. This is based on previous studies that the maximum tolerated dose (MTD) of eribulin is 1.5 mg/kg weekly in nu/nu mice (34), and the dose range of eribulin 0.05–1 mg/kg, which is below the MTD, intravenously or intraperitoneally induce tumor shrinkage in mouse xenograft models of different tumor types (35). In addition, pharmacokinetic studies of eribulin in mice (36) and in human phase I trials (37) suggest that eribulin 1 mg/kg in mice achieves area pharmacokinetic parameters that is within that of 1.4 mg/m^2^ in human (36, 37). As shown in Supplementary Fig. S1, when receiving eribulin at 1 mg/kg weekly x 3, tumor shrinkage was observed for WHIM29, WHIM10, and WHIM34, which were categorized as sensitive to eribulin. WHIM6, WHIM3, and WHIM4 had continued tumor growth, therefore considered relatively resistant to eribulin. WHIM52 and WHIM12 were classified as having stable disease because we did not observe any tumor shrinkage or growth.

### Improved Antitumor Effect with the Addition of Copanlisib to Eribulin

**FIGURE 2:**
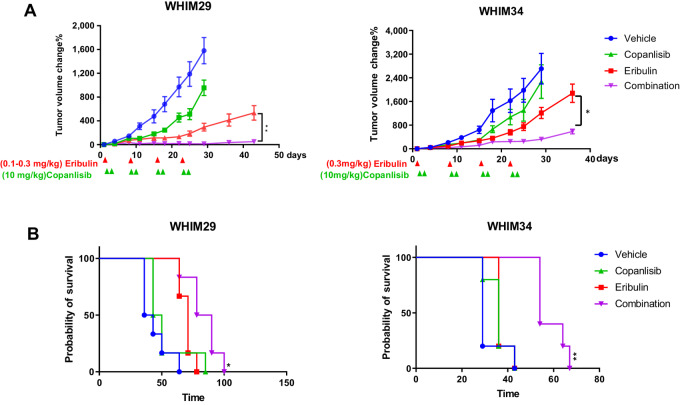
Tumor growth response to eribulin and copanlisib, either alone or in combination in WHIM29 and WHIM34. **A,** Tumor volume changes over time compared with that of day 1 after receiving either vehicle, eribulin (0.3 mg/kg i.p. on day 1 of each week × 4, except that 0.1 mg/kg eribulin was administered for the first two doses in WHIM29), copanlisib (10 mg/kg i.v. on days 2 and 3 each week × 4), or the combination of copanlisib and eribulin at the same dosing (*n* = 5 per group). **B,** Kaplan–Meier survival duration (days) of tumor-bearing mice after receiving treatments indicated in A. *, *P* < 0.05; **, *P* < 0.01, comparing between groups received eribulin or the combination of eribulin and copanlisib.

We first tested the two PDX models (WHIM29 and WHIM34) that carry *PTEN* mutations for their tumor growth response to copanlisib and eribulin, either alone or in combination. Tumor-bearing mice for each PDX model were randomly assigned to receive either vehicle, copanlisib, eribulin, or copanlisib plus eribulin for 4 weeks (*n* = 5 mice each treatment group, with two tumors per mouse). The combination therapy was most effective in reducing tumor growth (Fig. 2A) and prolonged survival (Fig. 2B) when compared with each single agent in both PDX models. The treatments were well tolerated. Body weights of mice treated with the combination therapy did not differ from those that received single agents (Supplementary Fig. S2).

We then screened the additional six PDX models for their response to the combination of eribulin and copanlisib. Tumor-bearing mice were treated with either vehicle, eribulin, copanlisib, or the combination of eribulin and copanlisib (*n* = 2 mice in each treatment group, with two tumors per mouse). The combination of eribulin and copanlisib was consistently effective in controlling tumor growth across the PDX models (Supplementary Fig. S3).

### Treatment-induced Proteomic Changes Indicate Target Inhibition by Copanlisib and Eribulin

**FIGURE 3:**
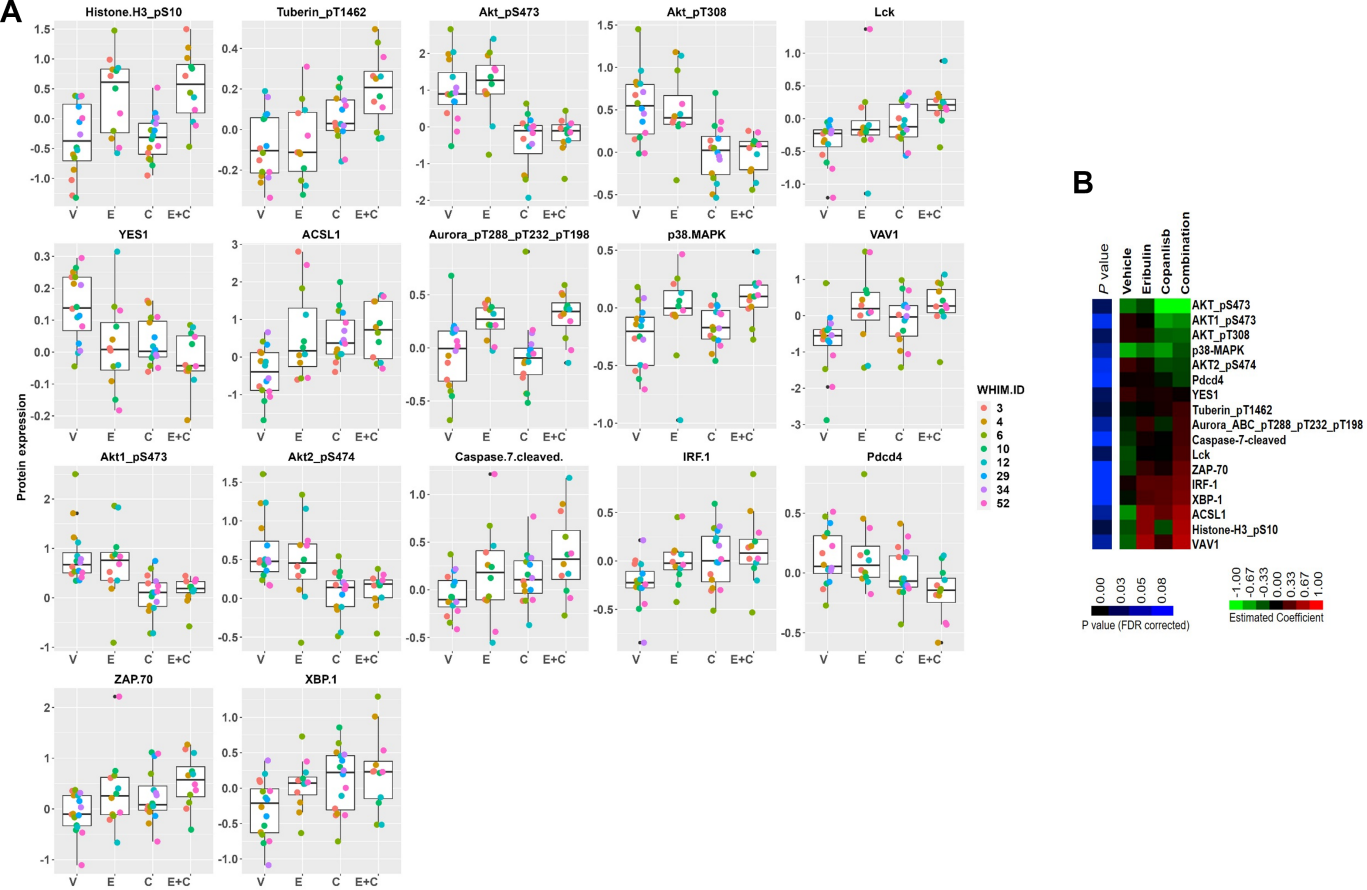
Expression of protein markers significantly altered by treatment with eribulin in combination with copanlisib in all PDX models. **A,** Expression of RPPA protein markers significantly altered, with FDR-adjusted *P* value <0.1, comparing treatment with the combination of eribulin and copanlisib versus vehicle are shown for individual tumors from each PDX model at the completion of 3–4 weeks of treatment with either vehicle, eribulin, copanlisib, or eribulin plus copanlisib. Note that WHIM34 and WHIM29 tumors treated with eribulin or eribulin in combination with copanlisib were missing in the analysis due to insufficient quantity of tumors at the completion of treatment. **B,** Heat map of estimated coefficient for FDR significantly altered markers (*P* < 0.1) comparing copanlisib plus eribulin versus vehicle.

**FIGURE 4:**
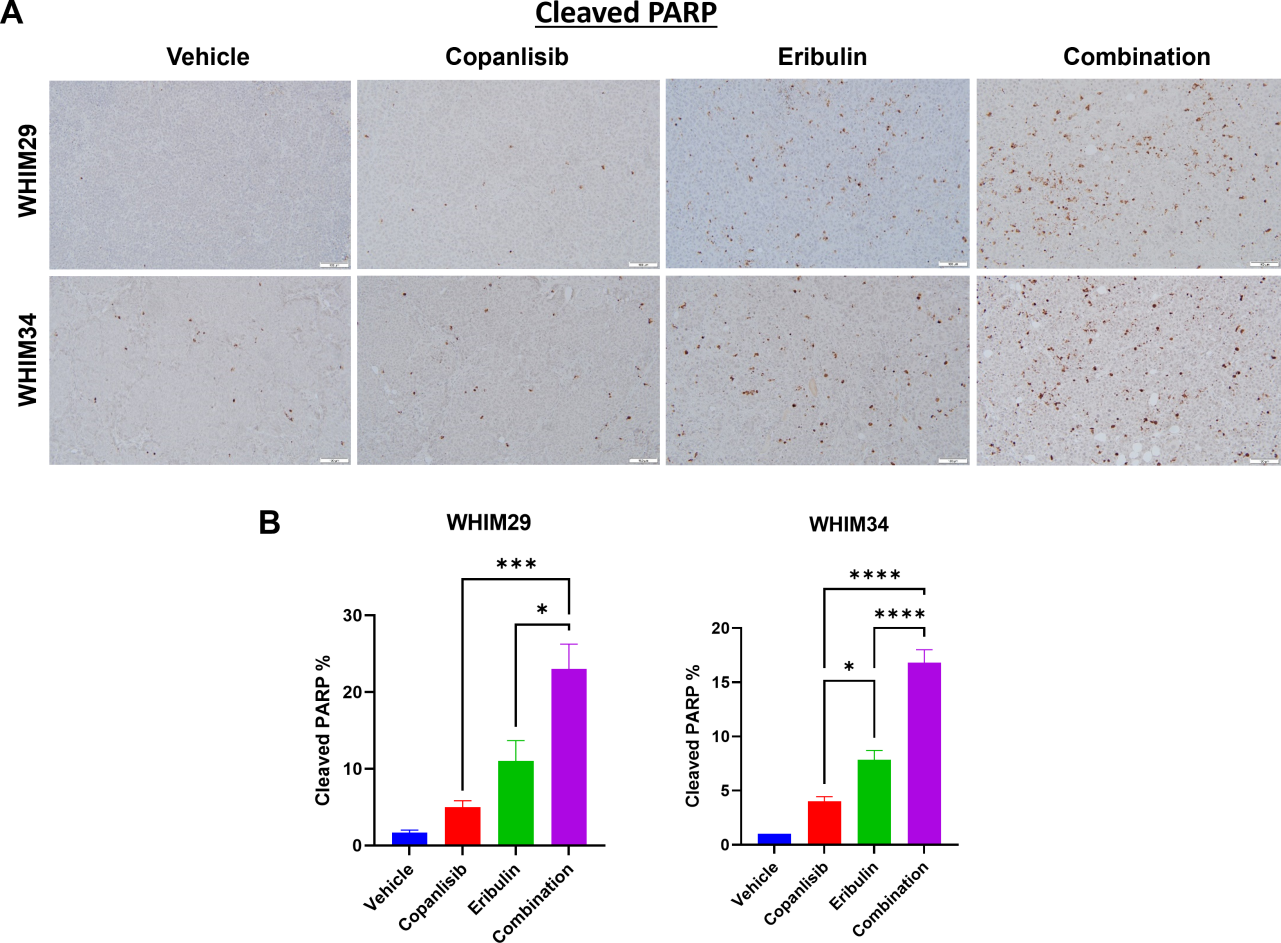
Apoptotic induction following treatment with eribulin and copanlisib, either alone or in combination by IHC. Representative IHC pictures (**A**) and quantifications of IHC scores (**B**) of cleaved PARP on WHIM29 and WHIM34 PDX tumors harvested on day 3 following treatment with either vehicle (day 1), eribulin (day 1), copanlisib (days 1 and 2), or the combination, and FDG PET 4 hours after treatment on day 2.

To assess target inhibition, mice were sacrificed with tumors harvested at the completion of 3–4 weeks therapy, 2 hours after the last treatment, followed by RPPA analysis of a 380-antibody panel at the MD Anderson RPPA core. Administration of copanlisib was associated with a significant reduction in PI3K pathway activity, demonstrated by significantly decreased levels of phosphorylated AKTs (Supplementary Table S3) while administration of eribulin was associated with increased mitotic markers such as phosphorylated histone H3 and Aurora kinases (Supplementary Table S4). Compared with vehicle, the combination of eribulin and copanlisib induced significant changes (FDR corrected *t* test *P* value <0.1) in the levels of 17 proteins (Supplementary Tables S5 and S6). Figure 3 shows the Box plots (Fig. 3A) and the Heatmap (Fig. 3B) of protein expression levels by treatment arm, of the 17 significant protein markers. The combination therapy significantly reduced AKT phosphorylation and upregulated mitotic markers as expected (Fig. 4; Supplementary Tables S5 and S6). In addition, the combination therapy significantly upregulated markers of apoptosis (cleaved caspase 7) and stress signaling p38 MAPK, as well as potential modulators of immune response (IRF-1, XBP-1, Lck, ZAP-70; Supplementary Tables S5 and S6). Significantly increased apoptotic induction following the combination of eribulin and copanlisib compared with that of each agent alone was confirmed by IHC of cleaved PARP on PDX tumors collected 48 hours after drug therapy in WHIM29 and WHIM34 (Fig. 4A and B).

**FIGURE 5:**
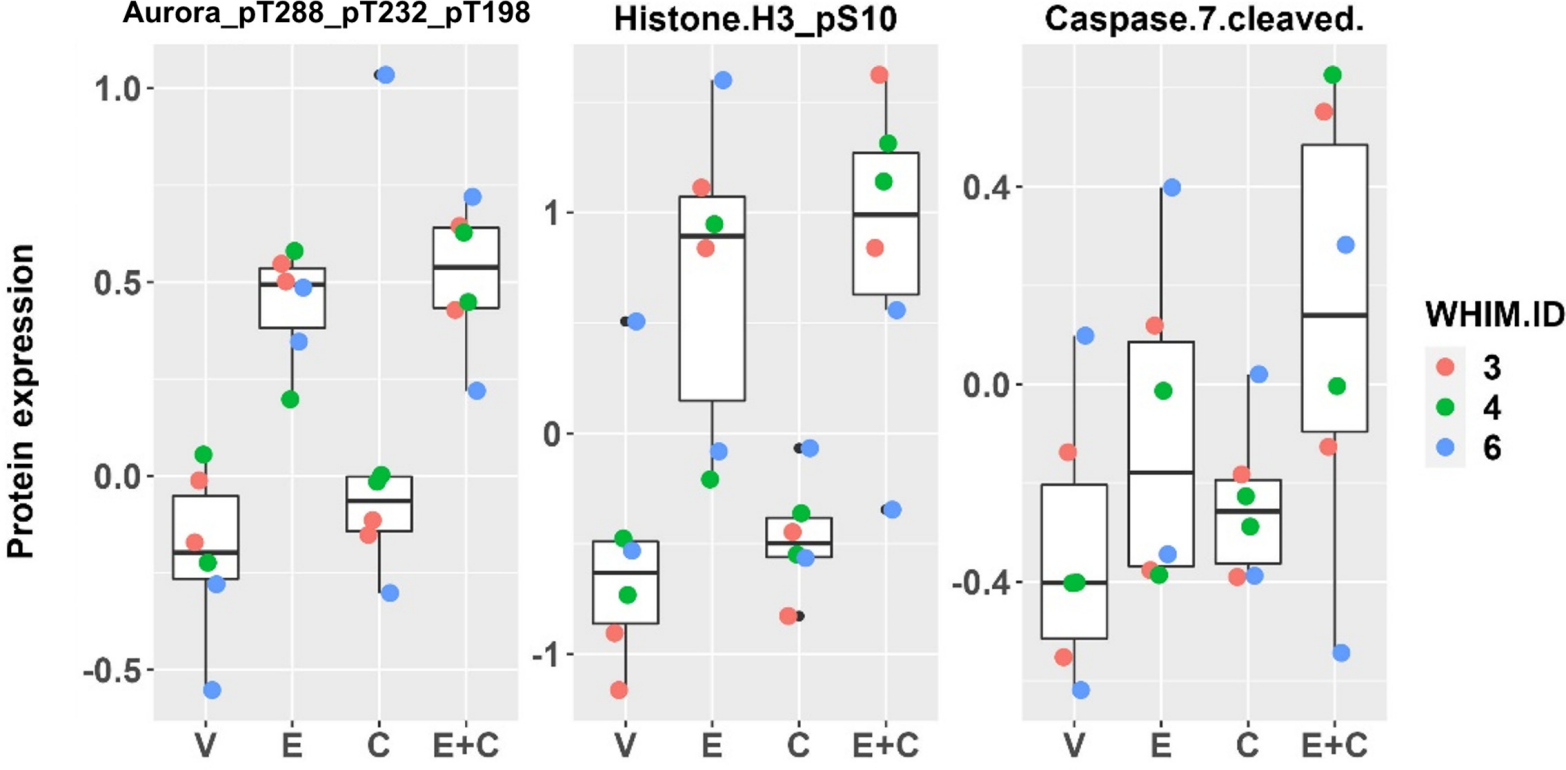
Expression of protein markers significantly altered by treatment with eribulin in combination with copanlisib in three eribulin-resistant models (WHIM3, WHIM4, and WHIM6). Expression of indicated RPPA protein markers are shown. Aurora_pT288_pT232_pT198, and Histone H3_pS10, were significantly upregulated, with FDR-adjusted *P* value <0.1, at the completion of 3–4 weeks of treatment with the combination of eribulin and copanlisib versus vehicle. Caspase 7 cleaved was upregulated following combination therapy, although did not reach statistical significance.

To examine the underlying molecular effect induced by the addition of copanlisib to eribulin in eribulin-resistant PDX models (WHIM3, WHIM4, and WHIM6), we analyzed the posttreatment RPPA data separately in this group and performed LIMMA differential analysis across treatments. We observed that Aurora_pT288_pT232_T198 and Histone H3_pS10, which reflect mitotic inhibition, were significantly increased following combination therapy compared with vehicle (FDR-adjusted F test *P* < 0.1; Fig. 5; Supplementary Table S7). These markers did not change significantly following eribulin monotherapy (Supplementary Table S7). The enhanced mitotic inhibition with the combination therapy was confirmed by IHC of pHistone H3 (Supplementary Fig. S4). Caspase 7_cleaved was increased following combination therapy, although this did not reach the defined statistical significance (Fig. 5; Supplementary Table S7). These data suggest that the addition of PI3K inhibitor enhanced the antimitotic effect of eribulin, leading apoptotic induction.

### Copanlisib Inhibited Eribulin-induced PI3K Pathway Signaling Activation

**FIGURE 6:**
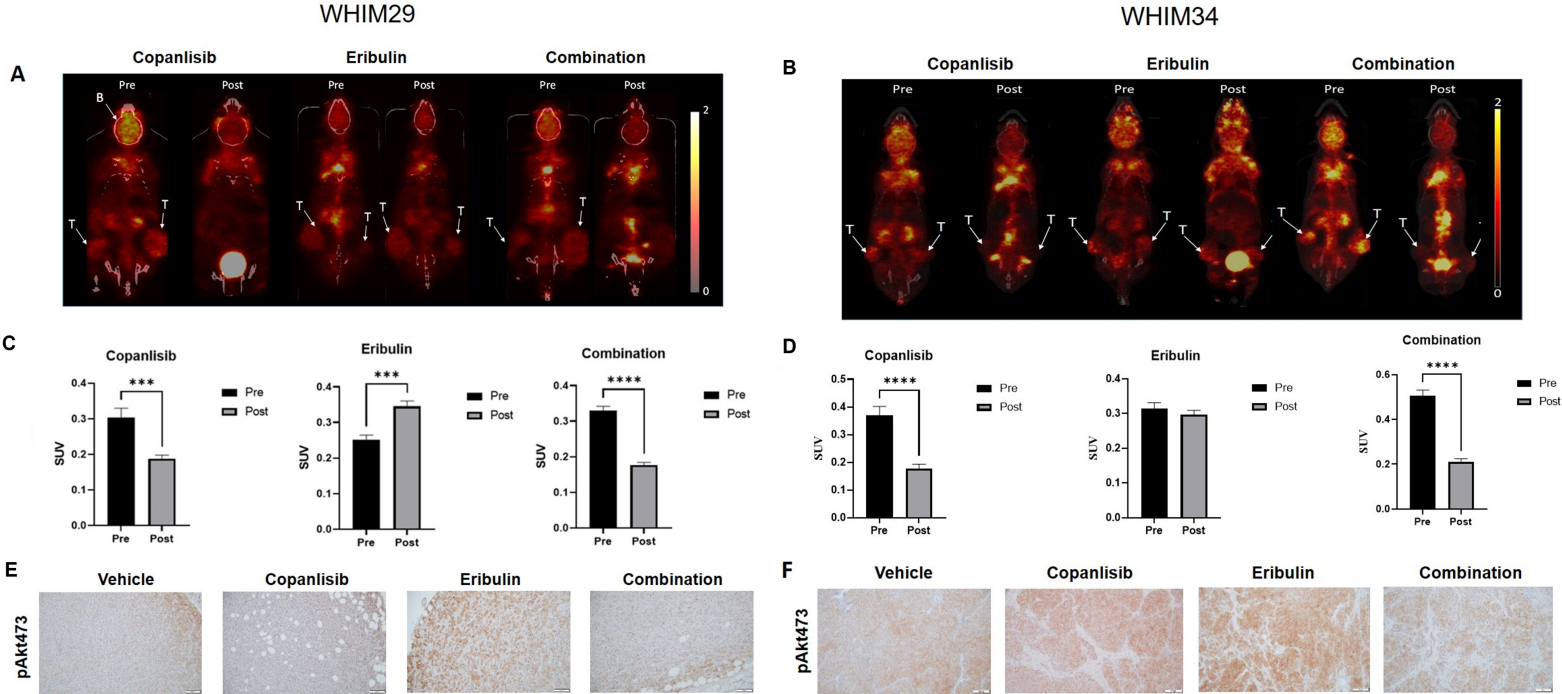
Treatment induced changes in FDG uptake by PET imaging and in the levels of pAKT by IHC in WHIM29 and WHIM34. Representative images of FDG PET scan of tumor-bearing mice performed pretreatment and posttreatment (on day 2, 4 hours following drug dosing) with either vehicle, copanlisib (10 mg/kg i.v., on day 1, and on day 2), eribulin (0.2 or 0.3 mg/kg i.p., on day 1), or the combination of eribulin and copanlisib for WHIM29 (**A**) and WHIM34 (**B**). Quantification of average SUV by FDG PET scan of tumor-bearing mice performed pretreatment and posttreatment with either copanlisib, eribulin, or the combination of copanlisib and eribulin in WHIM29 (*n* = 5; **C**) and WHIM34 (*n* = 5; **D**). ***, *P* < 0.001; ****, *P* < 0.0001. Representative IHC images for pAKT473 and cleaved PARP on tumor tissue sections harvested on day 3, following treatment with either vehicle, copanlisib (10 mg/kg i.v., on day 1, and on day 2), eribulin (0.2 or 0.3 mg/kg i.p., on day 1), or the combination of eribulin and copanlisib for WHIM29 (**E**) and WHIM34 (**F**).

FDG-PET is a clinically available noninvasive functional imaging approach to assess tumor glucose metabolism; therefore, an attractive biomarker in assessing the pharmacodynamic effect of PI3K inhibitor therapy. We performed FDG-PET scans for WHIM29 and WHIM34 tumor-bearing mice before and after 2 days of treatment with copanlisib and eribulin, alone or in combination. As shown in Fig. 6, copanlisib significantly reduced FDG SUV in both PDX tumors as expected (Fig. 6A–D). Interestingly, eribulin increased FDG uptake, which was reserved by the addition of copanlisib in WHIM29 (Fig. 6A and C). The FDG uptake changes corresponded to the changes in pAKT by IHC (Fig. 6E and F). Incidentally, we observed that copanlisib reduced FDG uptake in the brain (Fig. 6A and B), suggesting central nervous system availability of the drug in these mice.

## Discussion

There is a significant unmet clinical need for novel therapeutic approaches for TNBC. As chemotherapy is the mainstay of systemic therapy for TNBC, strategies that improve chemotherapy efficacy have the potential to improve patient outcomes. Eribulin is a common chemotherapy agent administered in the setting of progression on prior anthracycline- and taxane-based regimens. On the basis of studies from our lab and others demonstrating that PI3K inhibition enhances eribulin efficacy in TNBC (18, 38), we tested whether the pan-PI3K inhibitor copanlisib could improve tumor response to eribulin in a panel of TNBC PDX models. We demonstrated that copanlisib treatment significantly reduced PI3K/AKT/mTOR signaling and the combination of eribulin and copanlisib was broadly active against TNBC PDX models, regardless of PI3K pathway alterations, PTEN status, or sensitivity to eribulin monotherapy.

PI3K pathway inhibitors are attractive approaches to enhance chemotherapy efficacy in TNBC due to the high frequency of aberrant activation (2, 3). Previous studies have explored PI3K pathway inhibitors in combination with taxane regimens, however with mixed results (39, 40). Two randomized phase II trials have reported promising progression-free survival (PFS) advantages by adding AKT inhibitors to paclitaxel as first-line therapy for metastatic TNBC (39–41). In the LOTUS trial, the addition of the AKT inhibitor ipatasertib versus placebo, to paclitaxel significantly improved median PFS from 4.9 to 6.2 months [HR, 0.6; 95% confidence interval (CI), 0.37–0.98; *P* = 0.037; ref. 39]. The benefit of ipatasertib appeared to be more pronounced in patients with PI3K pathway alterations. Similarly, in the phase II PAKT trial, the addition of the AKT inhibitor capivasertib versus placebo resulted in a robust improvement in PFS in the *PIK3CA/AKT1/PTEN* altered group (capivasertib vs. placebo: 9.3 vs. 3.8 months, two-sided *P* = 0.01), as compared with the intention-to-treat cohort (capivasertib vs. placebo: 5.9 vs. 4.2 months) and *PIK3CA/AKT1/PTEN* unaltered tumors (capivasertib vs. placebo: 5.3 vs. 4.4 months, two-sided *P* = 0.61; ref. 40). However, the phase III IPATunity130 trial that enrolled 255 patients with locally advanced unresectable or metastatic TNBC harboring at least one alteration in *PIK3CA/AKT1* or *PTEN*, reported no significant difference in PFS between the ipatasertib arm and the placebo arm (7.4 vs. 6.1 months, HR, 1.02; 95% CI, 0.71–1.45; *P* = 0.9237; ref. 41).

PI3K inhibitors in combination with taxanes have also been explored in HER2-negative advanced/metastatic breast cancer. In the single-arm phase I/II trial of alpelisib plus nab-paclitaxel, 43 patients were enrolled (phase I, *n* = 13 and phase II, *n* = 30; ref. 42). More than two-thirds of the patients had received prior chemotherapy for metastatic disease, and one-third had received more than two lines of chemotherapy. The combination was found to be well tolerated and showed encouraging efficacy with an objective response rate of 59% including complete response in 7% of patients, and the median PFS was 8.7 months. Patients with tumor/circulating tumor DNA mutation demonstrated better PFS compared with those without mutation (11.9 vs. 7.5 months; HR, 0.44; *P* = 0.027). Compared with prediabetic/diabetic patients, those with normal metabolic status had longer PFS (12 vs. 7.5 months; *P* = 0.014). A phase III clinical trial (EPIK-B3, NCT04251533) is ongoing to assess alpelisib plus nab-paclitaxel as first- or second-line treatment for patients with advanced TNBC with *PIK3CA* mutation or PTEN loss.

We chose to study the PI3K inhibitor copanlisib in combination with eribulin because of their unique mechanisms of action. Eribulin is a non-taxane, synthetic macrocyclic ketone analog of halichondrin B which inhibits microtubule dynamics via suppression of microtubule polymerization and sequestration of tubulin into nonfunctional aggregates (13). Eribulin has been shown to inhibit EMT in preclinical models and in patients with breast cancer which was not observed with paclitaxel (17, 43, 44). As EMT may contribute to PI3K inhibitor resistance (45), eribulin could be a better chemotherapy partner than taxanes. Copanlisib is a potent pan-PI3K inhibitor that is administered intravenously, which may be advantageous compared with chronic daily dosing of orally administered PI3K inhibitors, as evidence is accumulating that complete target inhibition with intermittent dosing of PI3K inhibitors may be more effective and less toxic than continuous dosing regimens (19, 46, 47).

Similar to other PI3K inhibitors, single agent copanlisib was only partially effective in suppressing tumor growth (Fig. 2; Supplementary Fig. S3; refs. 18, 23). Interestingly, the added benefit of copanlisib to eribulin was independent of baseline PI3K pathway activity or genomic alterations. Although we cannot draw definitive conclusions based on the study of eight PDX models, the observation is consistent with the responses observed regardless of *PIK3CA* and PTEN status in the phase I trial of copanlisib monotherapy (20). A possible explanation is the activation of PI3K pathway signaling following treatment with eribulin, as demonstrated by increased FDG uptake on PET scan and AKT phosphorylation by IHC (Fig. 6). Our study is also consistent with previous reports that eribulin induced AKT phosphorylation in both preclinical models and in patients’ tumors and that the combination of eribulin and PI3K inhibitors were broadly effective in HER2-negative breast cancer preclinical models regardless of PI3K pathway alterations (48).

We observed that PI3K pathway activation, as measured by levels of pAKT by RPPA, was significantly correlated with mutations in *PIK3CA/PTEN* in the eight TNBC PDX models, except WHIM4 which has elevated pAKT level without mutations in the PI3K/PTEN pathway (Fig. 1). This is consistent with findings reported by Shi and colleagues in the analysis of baseline tumor biopsies obtained from patients with TNBC enrolled in the FAIRLANE trial of neoadjuvant Ipatasertib plus paclitaxel (49). The study demonstrated higher levels of AKT phosphorylation in tumors with genomic/protein alterations in PIK3CA/AKT1/PTEN and a subset of tumors without mutations in the pathway, likely due to alterations in upstream signaling pathways (49). The study also showed that Ipatasertib treatment led to a more pronounced downregulation of AKT/mTORC1 signaling in tumors with *PIK3CA/AKT1/PTEN* alterations. Further studies are needed to investigate whether this applies to PI3K inhibitors in clinical trials.

In eribulin-resistant PDX models, we observed significantly enhanced mitotic inhibition following combination therapy versus eribulin when compared with vehicle therapy, accompanied by an increased level of apoptosis (Fig. 5). This is consistent with our previous observation when combinating another PI3K inhibitor BKM120 with eribulin (18). However, unlike the previous report, we did not observe the effect on EMT marker N-cadherin following the combination of eribulin and copanlisib (Supplementary Table S6). The discrepancy may reflect the potential differences among PI3K inhibitors and/or biological heterogeneities among the TNBC PDX models.

FDG-PET is a clinically available noninvasive imaging modality that assesses glucose uptake in cancer cells. Changes in FDG uptake as early as 2 days after starting therapy has been shown to predict subsequent tumor volume responses to a PI3K inhibitor in HER2-positive breast cancer xenograft models (50). In addition, promising results demonstrating decreased FDG uptake on PET scan performed 2 weeks on alpelisib, a PI3K inhibitor, in correlation to PFS has been observed in early phase clinical trials for ER-positive breast cancers (51). Our data demonstrating reduced FDG uptake by PET performed 2 days after initiation of copanlisib or copalisib in combination with eribulin is in line with these results. These results highlight the potential translational utility of FDG-PET in monitoring response in patients with TNBC receiving this combination regimen, and importantly the pharmacodynamic effects of drugs in off-target organs. Additional studies evaluating the utility of FDG-PET as an early predictive biomarker in patients are needed.

Our preclinical PDX experiments demonstrated that the combination of copanlisib and eribulin is broadly effective for TNBC. These results provide the preclinical rationale for the clinical development of this combination in TNBC. This data led to a phase I/II trial of eribulin and copanlisib in patients with metastatic TNBC (clinicaltrials.gov #: NCT04345913). Unfortunately this trial was closed to enrollment early due to the strategic plan from Bayer to stop the clinical development of copanlisib due to the negative results from CHRONOS-4 trial in patients with relapsed follicular lymphoma. In CHRONOS-4, the addition of copanlisib to standard immunotherapy did not meet the primary endpoint of PFS https://www.bayer.com/en/us/news-stories/update-on-aliqopar. While we may not be able to validate the observed preclinical findings in clinical trials, our study provides important insights for future studies of other PI3K inhibitor or AKT inhibitors in combination with eribulin in TNBC.

## Supplementary Material

Supplementary Figure S1 Tumor growth response of TNBC PDX models to eribulin monotherapy

Supplementary Figure S2 Tolerability for the Combination of Eribulin and Copanlisib

Supplementary Figure S3 Tumor growth response to eribulin and copanlisib, either alone or in combination, in additional TNBC PDX models

Supplementary Figure S4 Mitotic inhibition following treatment with eribulin and copanlisib by IHC in 3 eribulin-resistant PDX models

Supplementary Table S1 Clinical Characteristics of Patient Derived Xenografts

Supplementary Table S2 Potential Actionable Mutations and Genomic Alterations in PI3K Pathway

Supplementary Table S3 Significantly Altered Levels of Proteins Following Treatment with Copanlisib

Supplementary Table S4 Significantly Altered Levels of Proteins Following Treatment with Eribulin

Supplementary Table S5 Significantly Altered Proteins Following Treatment with the Combination of Eribulin and Copanlisib including All 8 PDX Models

Supplementary Table S6 Statistical Analysis Comparing Changes in the Levels of Proteins by RPPA Among Different Treatment Groups for 8 PDX Models

Supplementary Table S7 Significantly Altered Levels of Proteins Following Treatment with the Combination of eribulin and copanlisib in Eribulin-resistant PDX Models (WHIMs 3, 4, and 6)
