# Supplementary material for: Evaluation of Copanlisib in Combination with Eribulin in Triple-negative Breast Cancer Patient-derived Xenograft Models
Source: Cancer Res Commun. 2024 Jun 5;4(6):1430–40. doi: 10.1158/2767-9764.CRC-24-0047 (PMC11152037; doi:10.1158/2767-9764.CRC-24-0047)
Supplement: Supplementary Figure S1 — Tumor growth response of TNBC PDX models to eribulin monotherapy [file crc-24-0047-s01.docx]

**
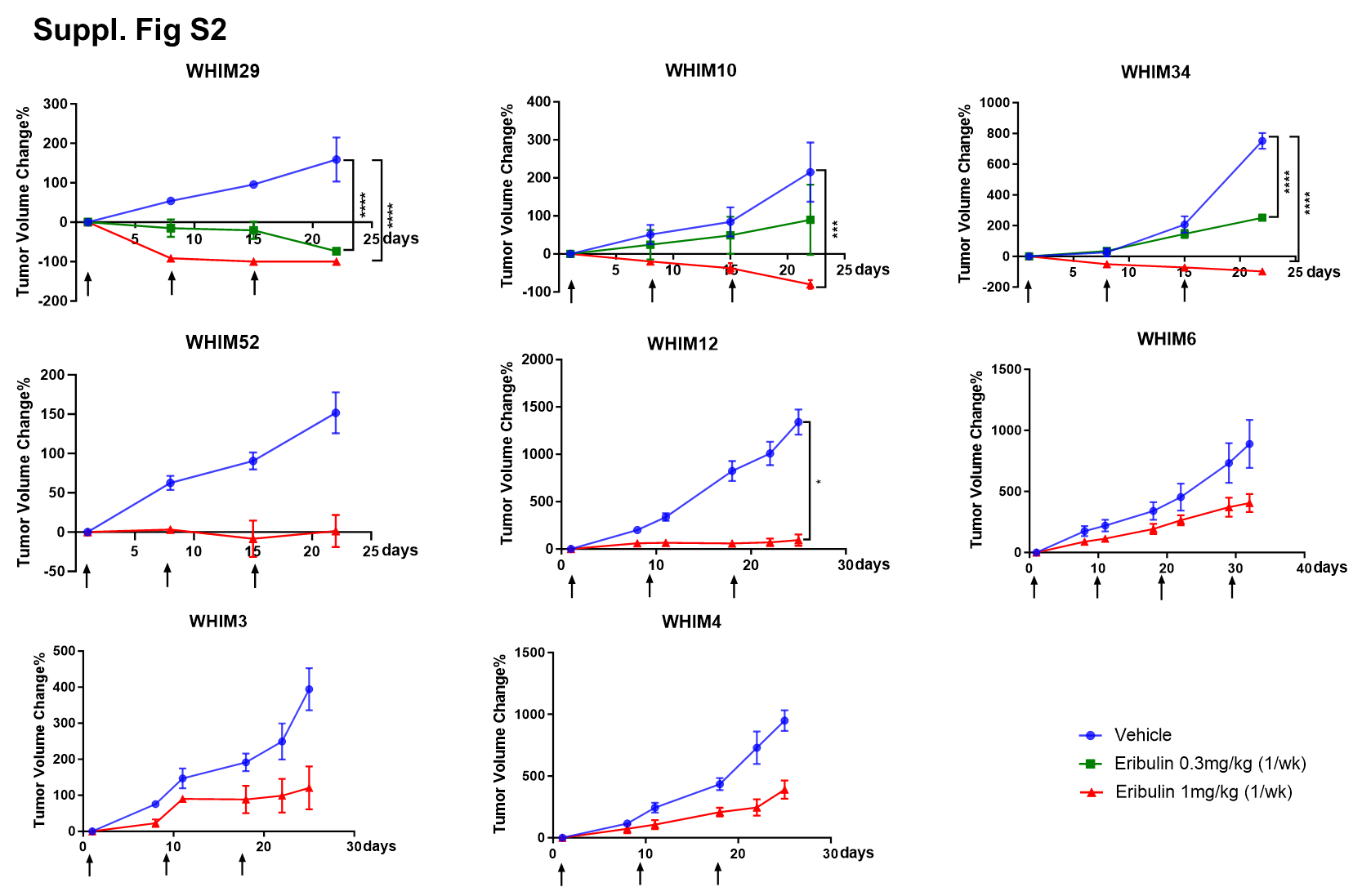
**

**Suppl. Fig S1. Tumor growth response of TNBC PDX models to eribulin monotherapy**

Tumor bearing mice for each PDX model were randomized to receive vehicle or eribulin (0.3 mg/kg and/or 1 mg/kg, IP on day 1 of each week) x 3 weeks when tumor volume reached 200 mm^3^. Percentage of tumor volume changes over time compared to that of day 1 were graphed for each PDX model.
