# Supplementary material for: Evaluation of Copanlisib in Combination with Eribulin in Triple-negative Breast Cancer Patient-derived Xenograft Models
Source: Cancer Res Commun. 2024 Jun 5;4(6):1430–40. doi: 10.1158/2767-9764.CRC-24-0047 (PMC11152037; doi:10.1158/2767-9764.CRC-24-0047)
Supplement: Supplementary Figure S2 — Tolerability for the Combination of Eribulin and Copanlisib [file crc-24-0047-s02.docx]

**
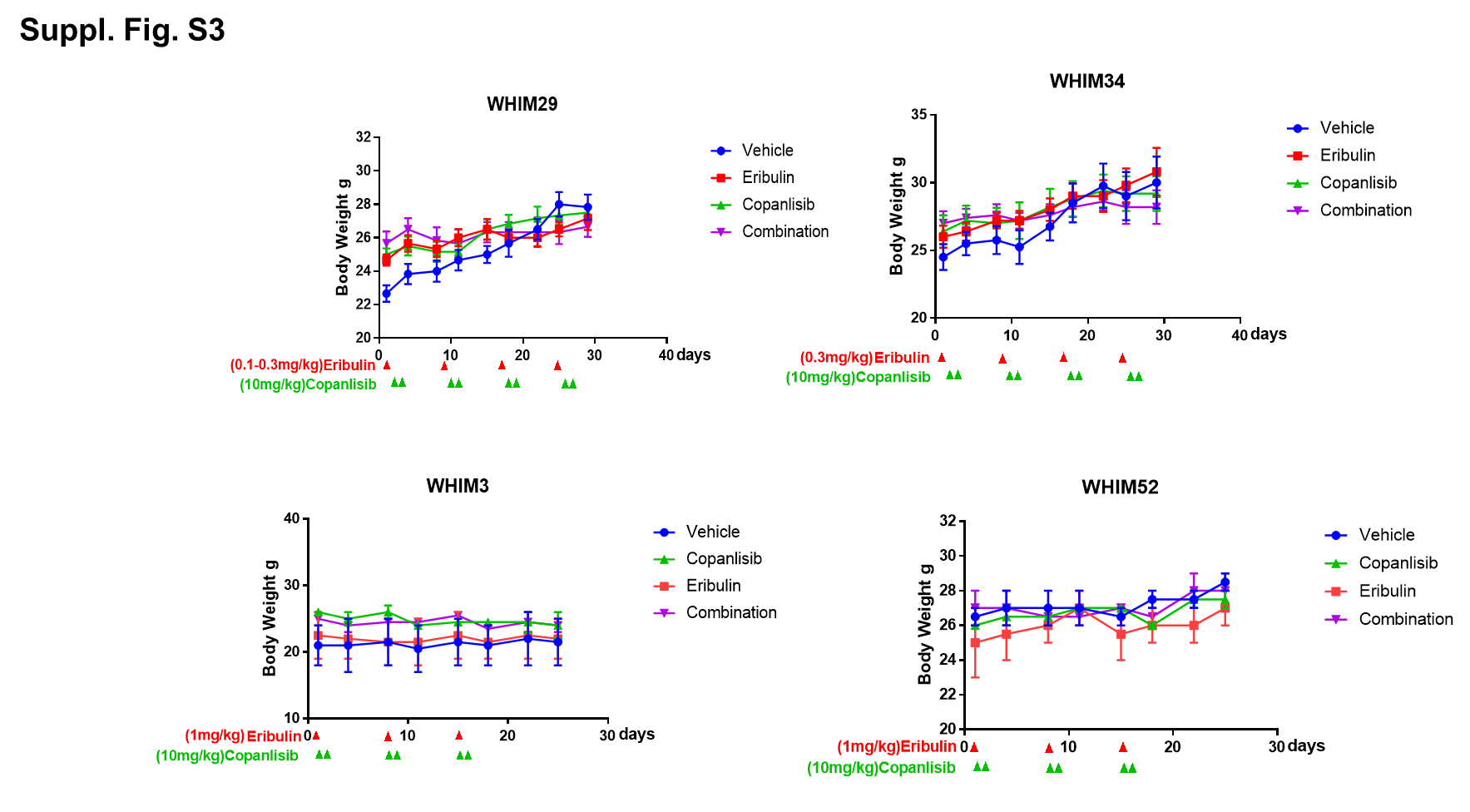
**

**Suppl. Fig S2. Tolerability for the Combination of Eribulin and Copanlisib**

Body weight was measured twice a week in tumor bearing mice treated as indicated. No weight loss was observed with either single agent or combination therapy with copanlisib and eribulin.
