# Supplementary material for: Evaluation of Copanlisib in Combination with Eribulin in Triple-negative Breast Cancer Patient-derived Xenograft Models
Source: Cancer Res Commun. 2024 Jun 5;4(6):1430–40. doi: 10.1158/2767-9764.CRC-24-0047 (PMC11152037; doi:10.1158/2767-9764.CRC-24-0047)
Supplement: Supplementary Figure S3 — Tumor growth response to eribulin and copanlisib, either alone or in combination, in additional TNBC PDX models [file crc-24-0047-s03.docx]

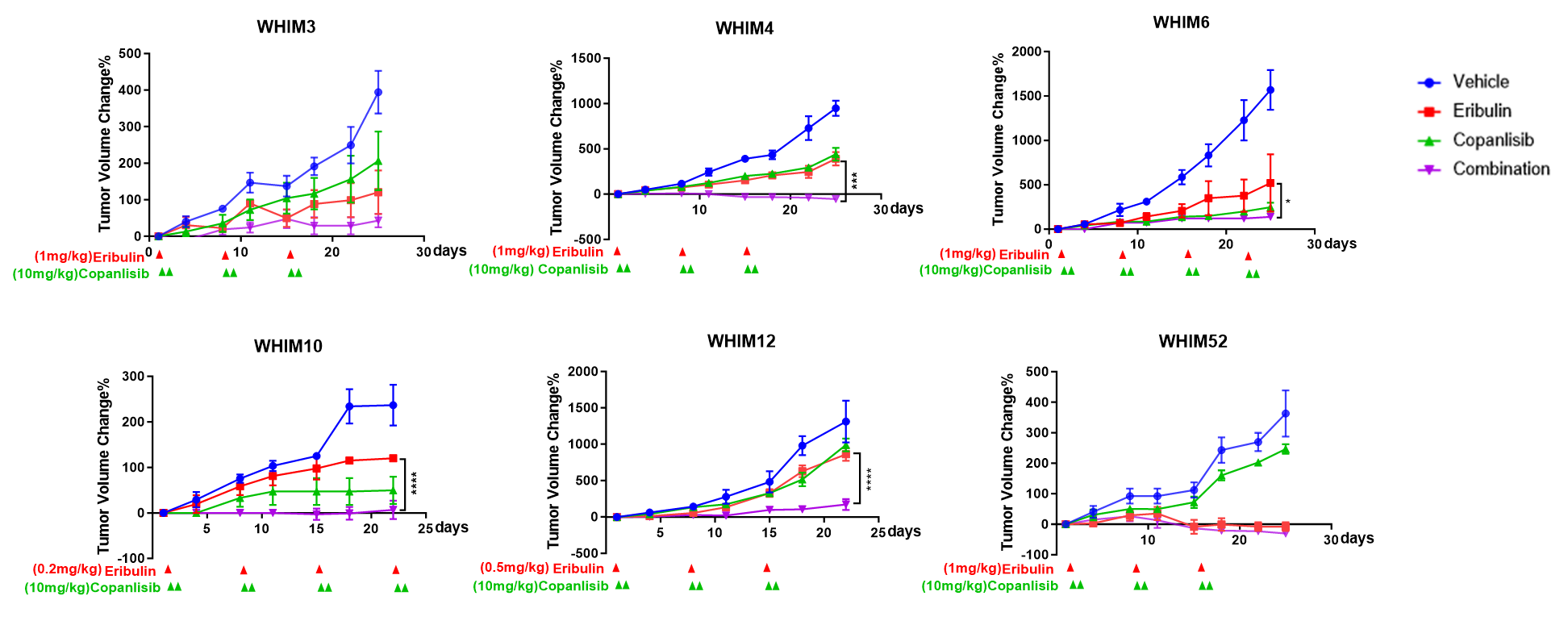


**Suppl. Fig S3. Tumor growth response to eribulin and copanlisib, either alone or in combination, in additional TNBC PDX models**

Tumor volume changes over time were graphed for indicated PDX model receiving treatment with either vehicle, eribulin, copanlisib, or the combination of copanlisib and eribulin (n=2 mice per group). *p<0.05, ***p<0.001, ****p<0.0001, comparing between groups received eribulin or the combination of eribulin and copanlisib.
