# Supplementary material for: Evaluation of Copanlisib in Combination with Eribulin in Triple-negative Breast Cancer Patient-derived Xenograft Models
Source: Cancer Res Commun. 2024 Jun 5;4(6):1430–40. doi: 10.1158/2767-9764.CRC-24-0047 (PMC11152037; doi:10.1158/2767-9764.CRC-24-0047)
Supplement: Supplementary Figure S4 — Mitotic inhibition following treatment with eribulin and copanlisib by IHC in 3 eribulin-resistant PDX models [file crc-24-0047-s04.docx]

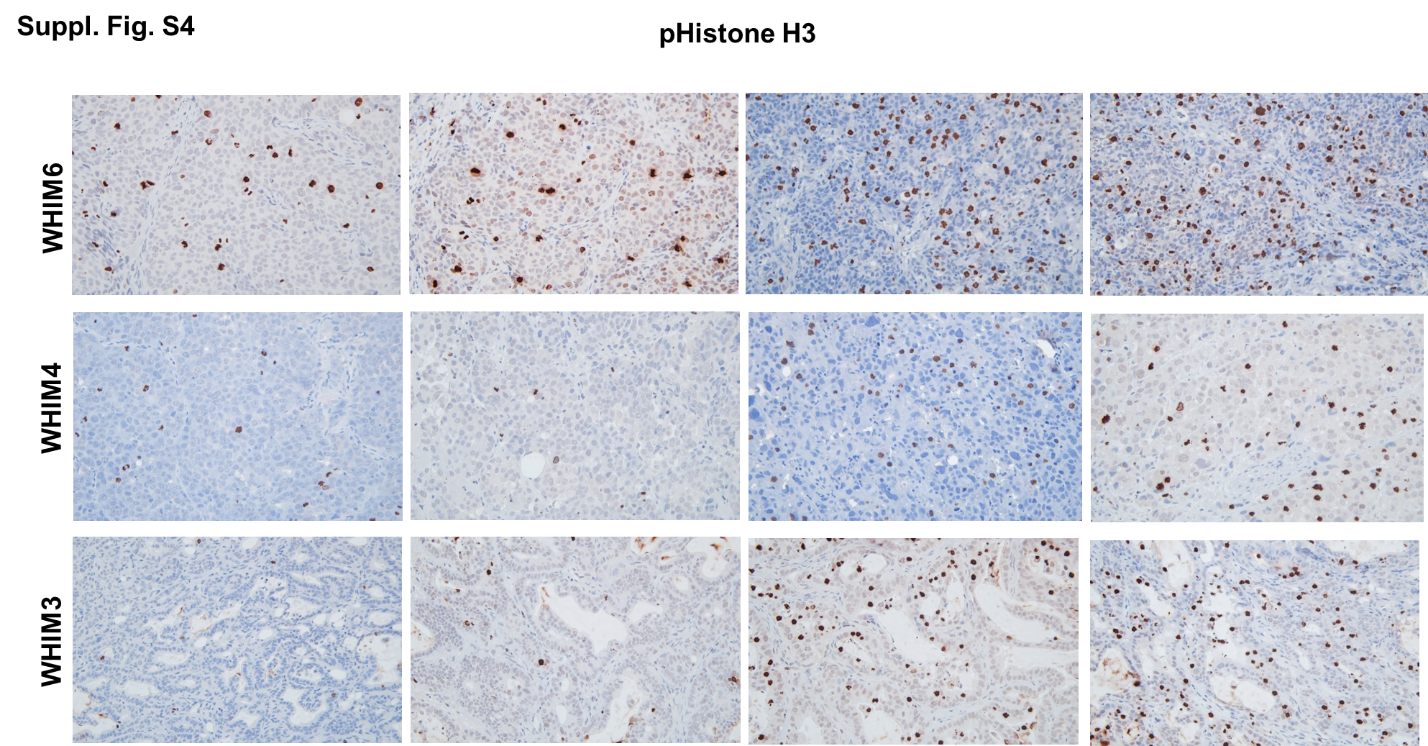


**Suppl. Fig S4. Mitotic inhibition following treatment with eribulin and copanlisib by IHC in 3 eribulin-resistant PDX models**

Representative IHC pictures of cleaved pHistone H3 on post-treatment PDX tumors harvested following 3-4 weeks of treatment with either vehicle, eribulin, copanlisib, or the combination, are shown.
