# Supplementary figures and images for: Evaluation of Copanlisib in Combination with Eribulin in Triple-negative Breast Cancer Patient-derived Xenograft Models
Source: Cancer Res Commun. 2024 Jun 5;4(6):1430–40. doi: 10.1158/2767-9764.CRC-24-0047 (PMC11152037; doi:10.1158/2767-9764.CRC-24-0047)

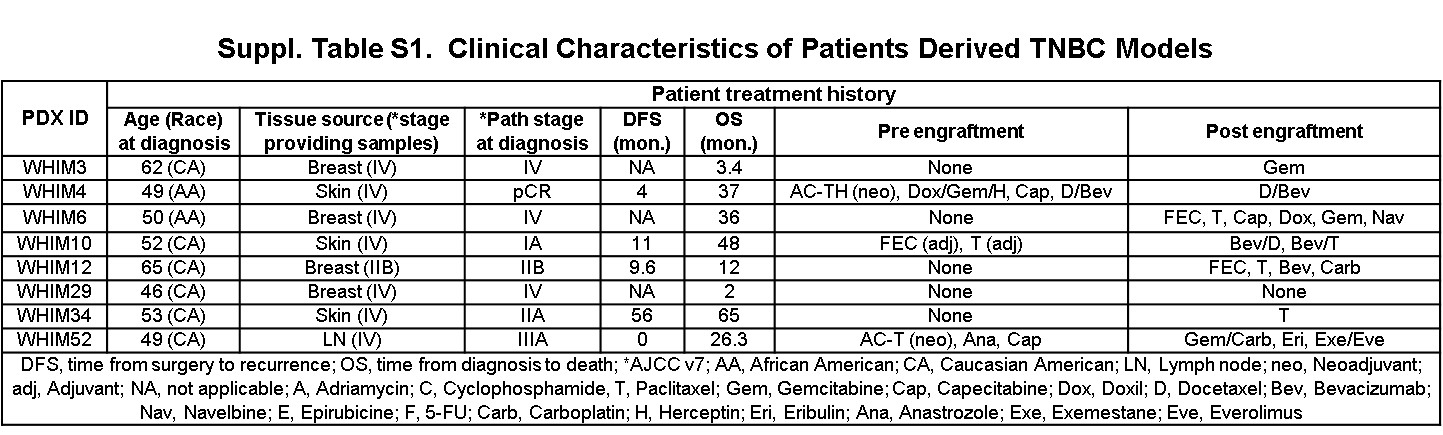

Supplement: Supplementary Table S1 — Clinical Characteristics of Patient Derived Xenografts [file crc-24-0047-s05.docx]

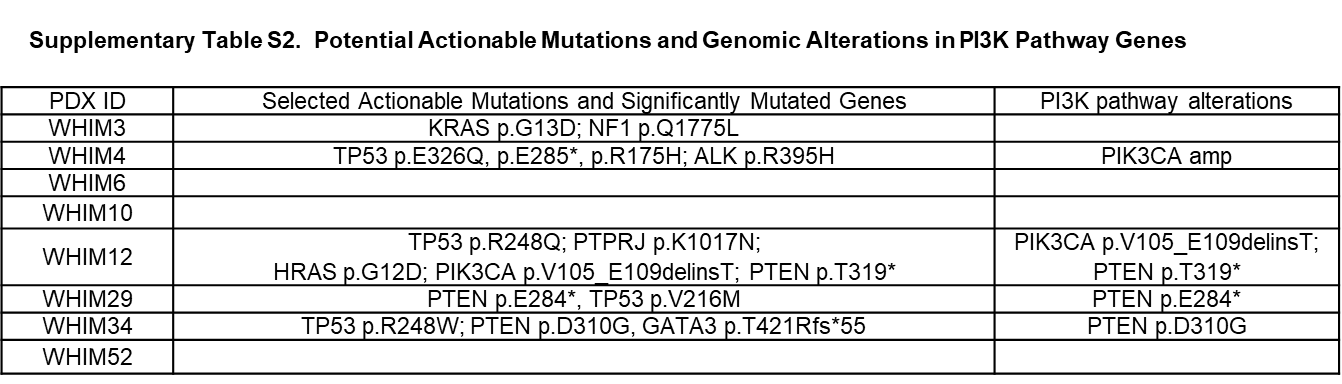

Supplement: Supplementary Table S2 — Potential Actionable Mutations and Genomic Alterations in PI3K Pathway [file crc-24-0047-s06.docx]

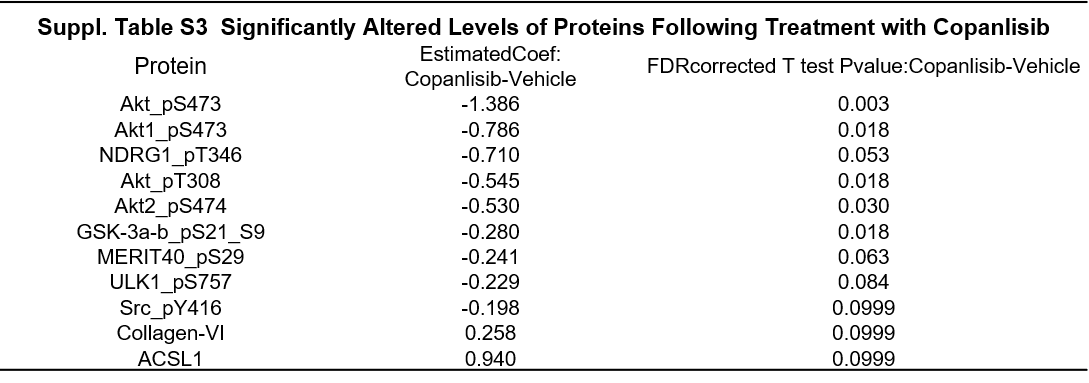

Supplement: Supplementary Table S3 — Significantly Altered Levels of Proteins Following Treatment with Copanlisib [file crc-24-0047-s07.docx]

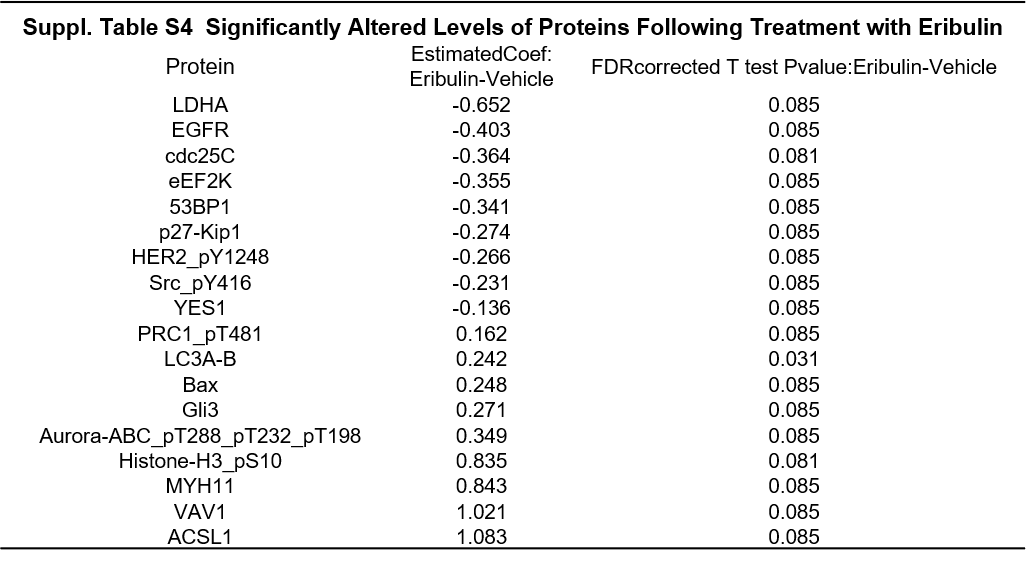

Supplement: Supplementary Table S4 — Significantly Altered Levels of Proteins Following Treatment with Eribulin [file crc-24-0047-s08.docx]

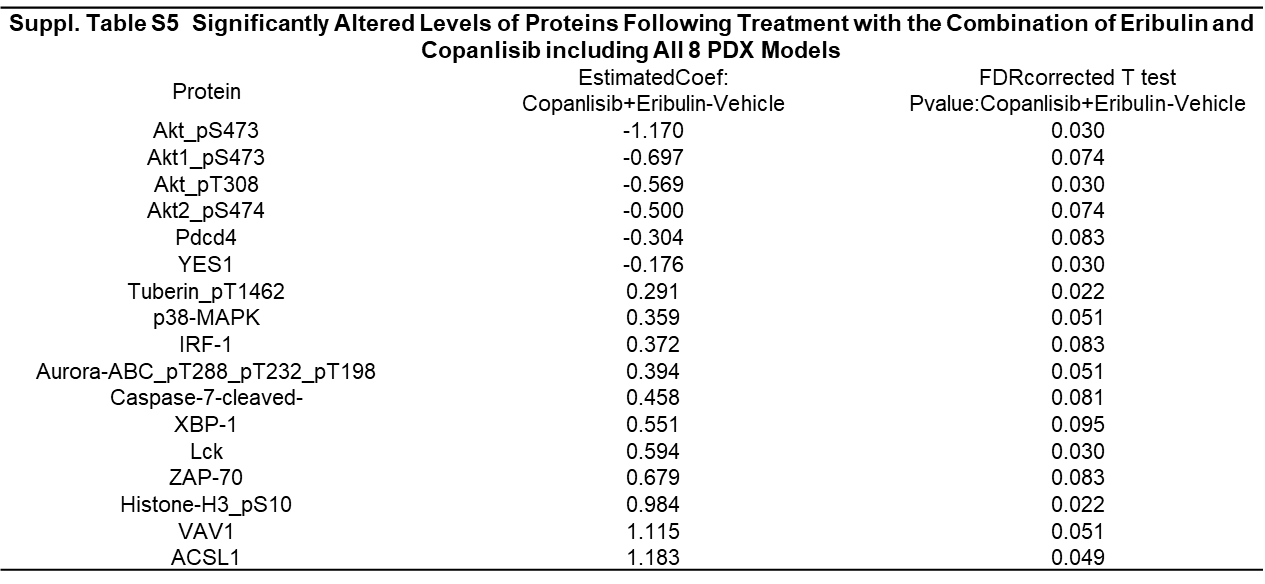

Supplement: Supplementary Table S5 — Significantly Altered Proteins Following Treatment with the Combination of Eribulin and Copanlisib including All 8 PDX Models [file crc-24-0047-s09.docx]

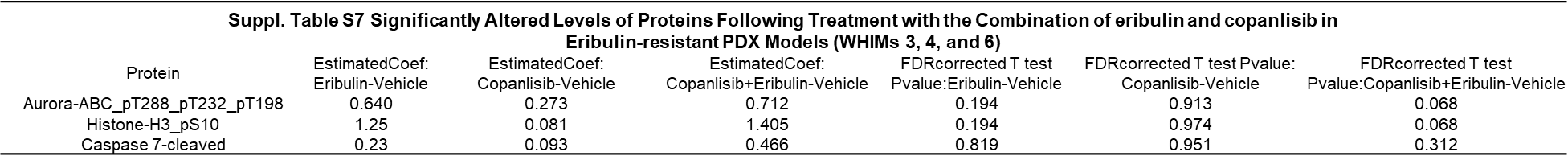

Supplement: Supplementary Table S7 — Significantly Altered Levels of Proteins Following Treatment with the Combination of eribulin and copanlisib in Eribulin-resistant PDX Models (WHIMs 3, 4, and 6) [file crc-24-0047-s11.docx]
